# Examining Approach and Avoidance Valences of the 3 X 2 Achievement Goal Types on an Engineering Student Sample: A Validity Approach

**DOI:** 10.3389/fpsyg.2021.628004

**Published:** 2021-09-09

**Authors:** Nathaniel Hunsu, Adurangba V. Oje, Andrew Jackson, Olanrewaju Paul Olaogun

**Affiliations:** ^1^College of Engineering, University of Georgia, Athens, GA, United States; ^2^College of Education, University of Georgia, Athens, GA, United States

**Keywords:** achievement goal, goal orientation, achievement motive, motivation, competence

## Abstract

Development of the 3 × 2 achievement goal questionnaire (AGQ) advanced approach and avoidance goals in three goal types within the achievement goal framework: task-, self-, and other-based. The purpose of the present study was to examine empirical support for the construct validity, reliability, and measurement invariance of factors on the questionnaire and compare model fit of the 3 × 2 configuration to other alternatives. In addition to validating some of the findings reported in earlier studies, especially the inclusion of task-based goal orientations, the study highlights a limitation and potential boundary of the 3 × 2 AGQ. While the 3 × 2 model was found to be structurally valid, we found multiple validity supports for a definition-based model of the AGQ scale, which does not differentiate between goal approach or avoidance. The study provides some indications that approach and avoidance goals can be indistinguishable to some respondents. Nonetheless, the scale was invariant across multiple groups making group comparison possible.

## Introduction

The achievement goal orientation theory was proposed in the later decades of the 20th century as a theoretical framework to explain and predict students’ goal-oriented achievement behaviors (Kaplan and Maehr, 2007). Conceptually, achievement goal constructs describe the purposes or motives for engaging in particular achievement behaviors (Maehr, 1989; Sparfeldt et al., 2015). The theory has evolved substantively from its initial roots within the achievement and competence-relevant motivation research literature (Elliot, 2005). Over the years, the achievement goal orientation (AGO) framework was popularized by the works of Elliot and colleagues on their achievement goal questionnaire (AGQ; e.g., Elliot and McGregor, 2001; Elliot et al., 2001; Harackiewicz et al., 2002; Murayama et al., 2009), and via those of Carol Midgley and others, via the Patterns of Adaptive Learning Scales (PALS; Midgley et al., 2000; Hackel et al., 2016).

Theorists described achievement goal orientations along a definition dimension based on the intrinsic nature of the competence-related behaviors that are associated with particular achievement goals and behaviors (Elliot and Thrash, 2001). By definition, achievement goals are said to be *mastery* oriented if achievement-related behaviors are impelled by a need to *attain* mastery, solely for mastery’s sake. Hence, mastery goals describe achievement-related behaviors that are motivated exclusively by a need to attain competence by *developing* or *attaining* mastery at a task or skill (Elliot et al., 2011). While, achievement goals are said to be *performance* oriented if achievement-related behaviors are impelled by the need to *demonstrate* competence relative to others. Performance goals describe achievement-related goals that are focused on demonstrating normative competence (Elliot and McGregor, 2001; Darnon et al., 2010). This initial characterization resulted in a *dichotomous model* which classified achievement behaviors as either mastery or performance oriented (Nicholls, 1989; Ames, 1992; Elliot, 2005).

Based on empirical data, however, theorists began to reconsider the sufficiency of the definition-based dimensionality of the achievement goal framework. Some theorists argued that the definition-based approach does not fully capture the valenced nature of the performance goal orientation observed in empirical literature (Middleton and Midgley, 1997; Skaalvik, 1997; Harackiewicz et al., 2002). Critics observed that individuals tended to *approach* competent performance, or *avoid* appearing incompetent. In response to this observation, theorists described performance achievement goals based on whether competence-related behaviors associated with achievement goals are positively or negatively referenced (Elliot and McGregor, 2001; Harackiewicz et al., 2002). This gave rise to a *trichotomous model* of the achievement goal theory that proposes *performance approach* and *performance avoidance* goals as separate goal types in addition to the mastery achievement goal type (Elliot and Harackiewicz, 1996; Cury et al., 2002; Pekrun et al., 2006; Payne et al., 2007).

Several studies provided empirical support to validate the approach and avoidance dimensions of performance goals across multiple contexts (Baranik et al., 2007; Nien and Duda, 2008). Meanwhile, other theorists suggested that the *trichotomous model* offered an incomplete picture of the achievement goal theory. They argued that people could lose skills and abilities they had acquired, or perform worse on a task than they did earlier (Elliot, 1999; Pintrich, 2000; Elliot and Conroy, 2005). Hence, their achievement-related behaviors may be regulated by a motivation to avoid becoming incompetent relative to the “absolute requirements of the task or one’s own pattern of attainment” (Elliot and McGregor, 2001, p. 509). As the definition-valence dimensions of the achievement goal theory gained acceptance, a 2 × 2 model that incorporated a mastery avoidance goal construct and expanded the earlier trichotomous model was proposed as a balanced conceptualization of the achievement goal theory. Hence, the 2 × 2 model describes achievement goals along a definition dimension as mastery and performance achievement goals. Along the valence dimension however, both mastery and performance goal types are bifurcated as *approach* or *avoidance* goals depending on whether they are positively or negatively oriented achievement behaviors (Elliot and McGregor, 2001). Mastery-approach goal orientation describes mastery achievement goals that are positively oriented toward attaining competence or mastery at a skill or task. Mastery-avoidance goal orientation describes achievement goals that focus on the need to avoid failing to attain mastery or competence at a task. Similarly, performance-approach goals define achievement goals that are positively oriented toward *demonstrating* competence, and performance-avoidance goals describe achievement goals that are focused on not appearing incompetent relative to others (Elliot, 1999; Baranik et al., 2010; Strunk et al., 2013).

### Advances in the Conceptualization of Achievement Goals

Both the mastery and performance goal constructs are conceptually described in terms of attaining competence (Elliot and McGregor, 2001; Elliot et al., 2011). Elliot et al. (2011) argued that competent performance may be evaluated exclusively relative to a task (i.e., based solely on the requirements of a task), to the self (i.e., based on intra-personal comparison of current and prior performances on a task), or to others (i.e., based on normative comparisons of one’s performance against others). During the early development of the achievement theory, the task and self-referenced standards for evaluating competence were subsumed within task-related achievement motives, and referred to the mastery goal orientation.

However, in recent development of the achievement goal theoretical framwork, Elliot et al. (2011) argued strongly that task and self-related achievement behaviors are distinct enough to be separated. They argued that, in the course of their many daily activities, people engage in tasks for the sake of the task alone. Whereas, there also are instances where individuals engage in tasks while being solely motivated, and consciously expending efforts, to improve on their own prior skill(s) or performance on the task. While their effort may be intrinsically tied to attaining mastery at the task, their predominant motive is on self improvement (i.e., relative to their past self). Hence, Elliot et al. (2011) proposed that the achievement goal framework be extended to reflect task-focused, self-focused, and other-focused achievement goal orientations (AGO) as separate goal types. They also proposed that each of these goal type maintain approach and avoidance valences.

**FIGURE 1:**
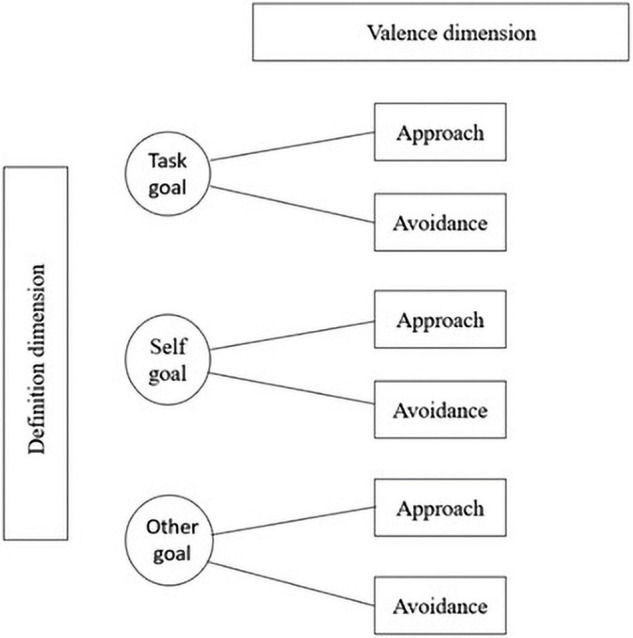
The 3 × 2 AGQ model. Competence *definition* dimension: task-focused (absolute requirements), self-focused (intra-personal standards), other-focused (inter-personal standards); Competence *valence* dimension: approach (positively oriented) and avoidance (negatively oriented behaviors).

In addition to arguing for separate task and self goal types, Elliot et al. (2011) proposed a *3* × *2 achievement goal model* that reflects approach-avoidance valences for the three goal types (Figure 1). By extending the achievement goal research landscape, the 3 × 2 model has the potential to further clarify and resolve inconsistencies in past research findings (Urdan and Kaplan, 2020). While the AGO frameworks highlighted above are the major theoretical conceptualization of the achievement goal framework to date, other AGO models (based on the AGQ and other scales) have also been explored in the literature. For example, Elliot et al. (2011) examined different achievement goal configurations in their study of the 3 × 2 AGQ. Similarly, researchers have also examined different antecedents and consequences of the different achievement goal orientations in various contexts (Elliot et al., 2011; Dinger et al., 2013; Schwinger et al., 2016).

### Past Validations of the 3 × 2 Model of the AGQ

A few studies have examined the validity of the 3 × 2 model of the AGQ, and compared it against alternative conceptualizations of achievement goal orientation (Elliot et al., 2011; Diseth, 2015; Lower and Turner, 2016; León-del-Barco et al., 2019). These studies reported finding better structural validity support for the 3 × 2 AGO framework than its alternatives. Although, some earlier studies reported finding very high correlations between the approach and avoidance valences of the three goal types. Lower and Turner (2016) reported correlations that ranged between *r* = 0.77 and *r* = 0.84; Elliot et al. (2011) found correlations ranging between *r* = 0.56 and *r* = 0.83 across two studies; Johnson and Kestler (2013) found correlations ranging between *r* = 0.86 and *r* = 0.89; and Diseth (2015) reported correlations that ranged between *r* = 0.45 and *r* = 0.79 within goal type. Participants in these studies included undergraduate students enrolled in psychology, sports, and education programs.

Scale factors with correlation greater than 0.85 are more difficult to distinguish from one another, calling into question their theoretical factor structure (Tabachnick and Fidell, 2001; Brown, 2006; Kline, 2010; Cohen et al., 2013). Kline (2010) argued that any two variables can hardly be deemed distinct if their correlations reach 0.90. Nonetheless, some theorists have proposed different rationale in defense of separating approach and avoidance valences for the different goal types. Murayama et al. (2011) argued that methodological artifacts (e.g., response bias, within-sample analysis, use of confirmatory factor analysis), sample (e.g., biased sample, change in goals), and environmental factors could be responsible for the high correlations that are often observed between approach and avoidance valences of achievement goals.

In addition to offering theoretical rationales, researchers have also sought empirical support for their position on separating approach and avoidance achievement goal valences. Elliot et al. (2011) examined relationships between different achievement goal types, and some of their antecedents and consequences. For example, using approach and avoidance temperaments as predictors, they found that approach temperament predicted approach valences for the task, self, other goal types, and avoidance temperament predicted avoidance goal valences. However, they also found that approach temperament predicted avoidance goal valences, and avoidance temperament predicted some approach goal valences. Nonetheless, the relationships they observed did not establish consistent patterns toward an unequivocal case for the uniqueness of approach and avoidance goal types.

Despite the case for their separation, the recurrence of very high correlations between the approach and avoidance valences of achievement goal types across multiple studies demonstrates a need to further examine how consistently students differentiate approach and avoidance goals in their achievement behaviors. Although high inter-factor correlations may not always indicate lack of distinctiveness between factors, it often raises concerns about discriminant validity (Henseler et al., 2009; Kline, 2010).

## The Present Study

In this study, we examined the factor structure of the 3 × 2 achievement goal model. Our first objective was to explore how well the factor structure of the 3 × 2 model holds up among a student sample with different a disciplinary training than those reported in earlier studies. To this end, we anticipated that our study would validate findings reported in earlier studies (Elliot et al., 2011; Diseth, 2015) and could highlight how robust the 3 × 2 AGO goal model is across diverse learning contexts. Inspection of the model included preliminary exploratory factor analysis (EFA) and confirmatory factor analysis (CFA), including the comparison of alternative AGO models.

Second, we evaluated construct (viz., convergent and discriminant) validity of the 3 × 2 goal factors. Some earlier studies have used relationships between different achievement goal factors and their antecedents and consequences (measured on other scales) to make inference about the construct validity of factors on the AGQ (Elliot et al., 2011; Dinger et al., 2013; Schwinger et al., 2016). However, we draw on the average variance extracted (AVE) and maximum shared variance (MSV), among goal factors on the AGQ itself, to assess their convergent and discriminant validity. Our purpose for evaluating indicators of convergent and discriminant validity was to determine whether we can empirically support keeping the approach and avoidance valences for the task, self and other achievement goal types separate when using the AGQ. Finally, we conducted invariance analysis to examine whether student groups interpret items on the AGQ in conceptually similar way. Respondent groups were based on several demographic characteristics, with comparisons made on the basis of several model fit indices.

## Materials and Methods

### Participants

Participants were invited to respond to the survey after institutional review board requirements and consents were obtained. Respondents included 437 undergraduate students enrolled in engineering programs at a large public research university in a southeastern state of United States. Participants self-identified as: male (72.5%) and female (27.5%); Caucasian (73%), Asian (10.5%), Black/African American (7.1%), Hispanic (5.6%), or another race (3.8%). Participant ages ranged from 17 to 41 years (*M* = 20.95; *SD* = 2.03). They were in the Sophomore (18.3%), Junior (52%), and Senior (29.7%) years of their degree programs.

### Measurement

The 3 × 2 version of the AGQ developed by Elliot et al. (2011) was administered to participants via the Qualtrics online survey platform. The questionnaire comprises 18 items that assess six achievement goal types – task-approach, task-avoidance, self-approach, self-avoidance, other-approach, other-avoidance. Each goal type is assessed by three items on a 5-point scale that ranged from 1 (being “Never or only rarely true of me”) to 5 (being “Always or almost always true of me”). Task-approach items were designed to assess students’ positively oriented task-focused achievement goal (e.g., “To know the right answers to the questions on the exams in this class”); while Task-avoidance items assessed negatively oriented task-focused achievement goal (e.g., “To avoid getting a lot of questions wrong on the exams in this class”). Self-approach items assessed positively oriented self-focused achievement goal (e.g., “To perform better on the exams in this class than I have done in the past on these types of exams”); Self-avoidance items assessed negatively oriented self-focused achievement goal (e.g., “To avoid doing worse on the exams in this class than I have done on prior exams of this type”). Other-approach items accessed positively oriented other-focused achievement goal (e.g., “To do better than my classmates on the exams in this class”); and Other-avoidance items assessed negatively oriented achievement goal focused on others (e.g., “To avoid doing worse than other students on the exams in this class”). The survey also included demographic items that captured information about participants’ gender, academic level, race, program of study, age, and whether they seriously considered going to graduate school.

## Data Analysis and Results

### Preliminary Analysis

We conducted preliminary analysis that included determining descriptive statistics and EFA to inspect how indicators on the AGQ scale load together onto unconstrained factors. The descriptive analysis and EFA were conducted using SPSS^®^ statistical software (version 26).

#### Descriptive Statistics and Correlations

Participant mean scores on the achievement goals ranged between 3.42 and 4.21 – both valences of the *task goal orientation* were rated the highest (*M* = 4.21). In contrast, the *other-approach* goal orientation was rated the least (*M* = 3.42). Correlations among achievement goal variables ranged between.163 and.989, *p* < 0.05. Within each goal type, the correlation between valences (approach and avoid) was high: *Task-approach* was positively associated with *task-avoidance* (*r* = 0.914, *p* < 0.05), *self-approach* was positively related to *self-avoidance* (*r* = 0.989, *p* < 0.05), and *other-approach* was positively associated with *other-avoidance* (*r* = 0.861, *p* < 0.05). The correlation between *other-approach* and *self-avoidance* was the least (*r* = 0.163, *p* < 0.05). In general, *other-approach* had the weakest relationship with other achievement goal orientations (*r* = 0.163 ∼0.342, *p* < 0.05). Descriptive statistics and correlation coefficients of the variables are shown in Tables 1, 2A respectively.

**TABLE 1:** Descriptive statistics of 3 × 2 achievement goal model (*N* = 437).

Variable	M	*SD*	Observed range
(1) Task approach	4.21	0.79	1.00–5.00
(2) Task avoidance	4.21	0.72	2.00–5.00
(3) Self-approach	3.99	0.80	1.00–5.00
(4) Self-avoidance	3.90	0.78	1.00–5.00
(5) Other approach	3.42	1.03	1.00–5.00
(6) Other avoidance	3.71	1.01	1.00–5.00

**TABLE 2:** **(A)** Correlations, composite reliability, convergent, and discriminant validity estimates for the baseline (3 × 2) AGO model.

	**Cronbach’s α**	**CR**	**AVE**	**MSV**	**Task-focused**		**Self-focused**		**Other-focused**	
					**Appr**	**Avoid**	**Appr**	**Avoid**	**Appr**	**Avoid**

Task Appr.	0.870	0.855	0.665	0.836	0.815					
Task Av.	0.837	0.834	0.626	0.836	0.914^*^	0.791				
Self Appr.	0.836	0.831	0.621	0.978	0.603^*^	0.689^*^	0.788			
Self Av.	0.774	0.712	0.46	0.978	0.523^*^	0.698^*^	0.989^*^	0.678		
Other Appr.	0.911	0.878	0.708	0.742	0.342^*^	0.319^*^	0.181^*^	0.163^*^	0.842	
Other Av.	0.915	0.905	0.761	0.742	0.327^*^	0.392^*^	0.310^*^	0.348^*^	0.861^*^	0.873

**(B)** Correlations, composite reliability, convergent, and discriminant validity estimates for the EFA-based AGO model.										

	**Cronbach’s α**		**CR**	**AVE**	**MSV**	**Task-focused**		**Self-focused**	**Other-focused**	

Task-focused	0.908		0.90	0.602	0.429	0.776				
Self-focused	0.894		0.871	0.536	0.429	0.655^*^		0.732		
Other-focused	0.935		0.919	0.657	0.148	0.385^*^		0.308^*^	0.810	

^*^Signifies that there a factor has a *p* < 0.01.

*Diagonal values are the square root of AVE.*

#### Preliminary EFA

An EFA was conducted to explore how many factors can be observed in our participants responses if no theoretical constraint were improsed on the data. Factors were extracted and rotated using Maximum Likelihood estimation technique and direct oblimin respectively. Bartlett’s test of sphericity suggested that the data was suitable for an EFA [χ^2^(153) = 2616, *p* < 0.001]. Kaiser–Meyer–Olkin (KMO) measure indicated there was an adequate sample for the analysis (0.853). An unconstrained EFA yielded three factors with Eigenvalues greater than 1. The three factors extracted explained 62.3% of the cumulative variance in participants’ response. Table 3 shows a pattern matrix that describes item loading, variance explained and internal reliability of the scale based on the EFA. Approach and avoidance items of each goal type loaded together, irrespective of their valences on the three factors extracted as the table shows.

**TABLE 3:** EFA pattern matrix of the rotated factor loadings for the 3 × 2 AGQ scale.

	Self-goals *Factor 1*	Other-goals *Factor 2*	Task-goals *Factor 3*
Self approach – Item 3	**0.901**	–0.017	–0.102
Self approach – Item 2	**0.809**	0.025	–0.083
Self avoidance – Item 3	**0.739**	–0.020	0.073
Self approach – Item 1	**0.676**	–0.006	0.024
Self avoidance – Item 2	**0.519**	–0.005	0.096
Self avoidance – Item 1	**0.370**	0.043	0.207
OAP_3	–0.067	**0.834**	0.032
Other Avoidance – Item 1	–0.178	**0.829**	0.012
Other Avoidance – Item 3	0.150	**0.829**	–0.138
Other Avoidance – Item 1	0.125	**0.694**	0.031
Other Avoidance – Item 2	0.191	**0.582**	0.002
OAP_2	–0.097	**0.576**	0.160
TSK approach 2	–0.141	0.013	**0.833**
TSK approach 3	–0.122	0.117	**0.793**
TSK_ approach 1	0.081	–0.018	**0.775**
TSK avoidance 2	0.253	–0.041	**0.612**
TSK avoidance 1	0.254	–0.042	**0.591**
TSK avoidance 3	0.279	0.000	**0.474**
Eigenvalue	7.37	2.41	1.398
Variance Explained (%)	40.95%	13.55	7.76%
Cronbach’s α	0.85	0.88	0.88

*Bold face denotes factor loading greater than 0.03.*

### Confirmatory Factor Analysis

Next, we conducted CFA to examine the structure of the 3 × 2 model and to compare it against alternative AGO models. Reliability, AVE-based validity analysis, measurement invariance and group comparisons were also conducted on the basis of these models. A cursory inspection showed that the data included 21 missing values (about 1.7%). The result of Little’s Missing Completely at Random (MCAR) test was significant, Chi-Square = 162.854, *df* = 87, *p* < 0.01, indicating that the data was not missing completely at random. Since missing values in the data was negligible (<5%), we did not use any advanced imputation method in treating missing value (Jakobsen et al., 2017), missing values was were treated using median replacement method.

Confirmatory factor analysis (*N* = 437) was conducted in SPSS^®^ AMOS software (version 26) based on a covariance matrix; factor solutions were estimated using maximum likelihood. The variances of latent factors specified on the scale were fixed to 1 to identify the model. Indices used in evaluating model fit included Comparative Fit Index (CFI), Tucker-Lewis Index (TLI), Root Mean Square Error of Approximation (RMSEA), and Standardized Root Mean Square Residual (SRMR). Adequacy of model fit was based on recommended threshold of fit indices – CFI ≥ 0.95, TLI ≥ 0.95, RMSEA ≤ 0.06, and SRMR ≤ 0.08 (Hu and Bentler, 1999). Some authors have suggested that a model with RSMEA ≤ 0.08 is acceptable (Hair et al., 2010).

#### 3 × 2 Achievement Goal Model

First we conducted CFA to examine the 3 × 2 model proposed by Elliot et al. (2011). The CFA result showed that the observed data supported the model. Each item load significantly as specified on the model and standardized factor loadings for all 18 items ranged between | 0.57| and | 0.97|. Similarly, the model yielded a good fit (χ^2^ = 340.16, df = 120, *p* < 0.01, CFI = 0.96, TLI = 0.95, RMSEA = 0.065, and SRMR = 0.05). These results were similar to those reported by Elliot et al. (2011). We observed very high correlations between the approach and avoidance valences of the Task (*r* = 0.914), Self (*r* = 0.989), and Other (*r* = 0.861) achievement goal types (Table 2A). High inter-factor correlations are often concerning for factor distinctiveness (Brown, 2006; Kline, 2010). Alternative AGO models tested in earlier studies (Elliot et al., 2011; Diseth, 2015; Mascret et al., 2015) were also examined.

#### Definition-Based Model From EFA Results

**FIGURE 2:**
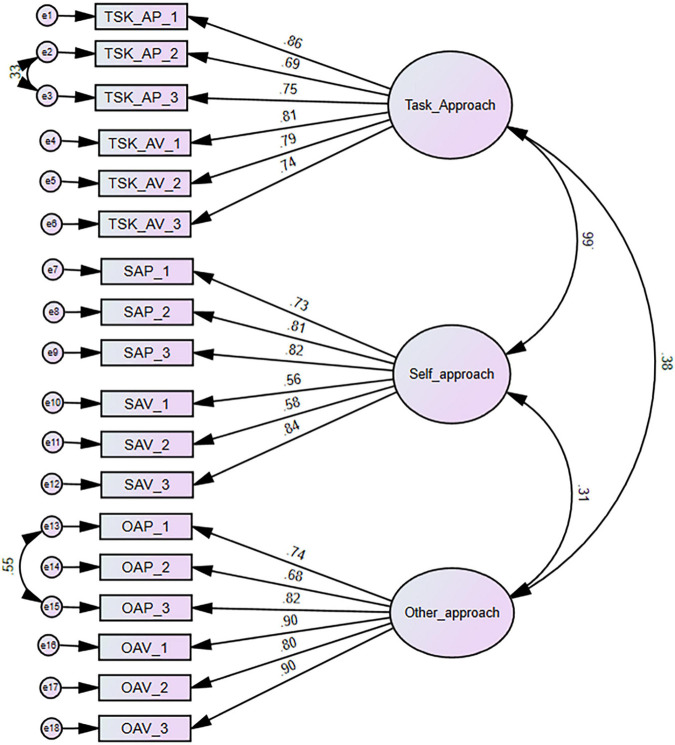
Definition-based AGO model based on EFA result.

Items on the approach and avoidance valences of each goal type were combined to create a three factor model consistent with the unconstrained model from our preliminary EFA. The model (Figure 2) was of definition-based achievement goals and was specified in response to high correlations observed between the approach and avoidance valences of the task-based, self-based, and other-based goal types. As such, each goal type comprised six indicators when their avoidance and approach indicators are combined. The initial fit of the model was marginal: χ^2^ = 580.92, df = 132 *p* < 0.01, CFI = 0.91, TLI = 0.90, RMSEA = 0.09. Modification indices suggested that two item pairs (2 and 3, and 13 and 15) be permitted to covary, which improved model fit significantly: χ^2^ = 420.39, df = 130, *p* < 0.01, CFI = 0.95, TLI = 0.93, RMSEA = 0.07, and SRMR = 0.05. Standardized factor loading for the all 18 ranged between | 0.56| and | 0.90|. The model was comparable to the 3 × 2 model.

#### 2 × 2 Achievement Goal Model

We specified a 2 × 2 (four-factor) equivalent to earlier AGO models including mastery- and performance-based definitions. In the first factor on the model, we combined indicators of the Task-approach and Self-approach goals. Indicators of Task- avoidance and Self-avoidance goals were also combined onto a second factor. Indicators of other-approach and other-avoidance goals were kept as separate latent factors. Model fit for the 2 × 2 model was unacceptable (χ^2^ = 980.61, df = 131, *p* < 0.01, CFI = 0.84, TLI = 0.81, RMSEA = 0.12, and SRMR = 0.08).

#### Trichotomous Achievement Goal Model

Next, we compared a trichotomous achievement goal model based on a past conceptualization of goal orientations (Elliot and McGregor, 2001; Elliot et al., 2011). In this model, indicators of task-based and self-based goal types (irrespective of their valences) were combined to form a single latent factor. The other-approach and other-avoidance goal types were kept as separate latent factors, as in the 2 × 2 model above. Model fit for the trichotomous model was unacceptable (χ^2^ = 985.46, df = 132, *p* < 0.01, CFI = 0.84, TLI = 0.81, RMSEA = 0.12, and SRMR = 0.08).

#### Dichotomous Achievement Goal Model

The final model simplified AGO to mastery- and performance-based factors. Indicators of task-based and self-based goal types were combined to form a single latent factor in this model. The other-approach and other-avoidance goal indicators were also combined to form a single latent factor. Model fit for the dichotomous model was unacceptable (χ^2^ = 1130.59, df = 134, *p* < 0.01, CFI = 0.81, TLI = 0.78, RMSEA = 0.13, and SRMR = 0.08).

#### Model Comparison Based on CFA

In addition to model fit statistics, Chi-square difference test was conducted, and we inspected Akaike information criterion (AIC) and Browne-Cudeck criterion (BCC) to compare competing models – lower values of AIC and BCC indicate better model fit (Vrieze, 2012). Results of all models are reported in Table 4. An inspection of the general fit statistics, AIC and BCC values of the models indicates that the baseline model was marginally better than that of the definition-based trichotomous model. However both models were significantly better than the other three models.

**TABLE 4:** Model fit comparisons of 3 × 2 and alternative achievement goal models.

Model	χ^2^	df	CFI	TLI	RMSEA	SRMR	Model Comparisons		
							Δχ2	AIC	BCC
3 × 2 (Baseline)	340.16	120	0.96	0.95	0.07	0.05	–	478.16	484.45
EFA, definition-based trichotomous	420.39	130	0.95	0.93	0.07	0.05	80.23	502.39	506.12
2 × 2	980.61	131	0.84	0.81	0.12	0.08	640.45	1060.61	1064.26
Trichotomous	985.46	132	0.84	0.81	0.12	0.08	645.3	1063.46	1067.01
Dichotomous	1130.59	134	0.81	0.78	0.13	0.08	790.43	1204.59	1207.96

*N = 437.*

*RMSEA, root mean square of approximation; SRMR, standard root mean square residual; TLI, Tucker Lewis Index; CFI, Comparative Fit Index; AIC, Akaike Information criterion; BCC, Browne-Cudeck criterion.*

*Δχ^2^, ΔCFI, ΔRMSEA, AIC, and BCC statistics compare the alternative model to the baseline model. All statistics differs significantly at the p < 0.01 level.*

### Reliability (α and CR) and AVE-Based Validity

We examined indicators of internal reliability and construct validity for the baseline (3 × 2) model and its definition-dimension-based trichotomous alternative. Coefficients of internal consistency for factors associated each model ranged between 0.77 and 0.94. The adequacy of using Cronbach alpha coefficient as a measure of internal reliability has been criticized because it is based on the assumption that scale items have equal factor loadings (Peterson and Kim, 2013). Alternatively, composite reliability (CR) or Omega coefficients are recommended as preferable indicators of internal reliability (Peterson and Kim, 2013; McNeish, 2018). Composite reliability coefficients of both models (reported on Tables 2A,B) were better than the acceptable threshold of 0.70 (Hair et al., 2010), though composite reliability coefficients on the definition-based model were much better than those of the 3 × 2 model.

An AMOS plugin developed by Gaskin and Lim (2016) was used to estimate average variance extracted (AVE) and maximum shared variance (MSV)—which were used together to infer convergent and discriminant validity of the 3 × 2 and the definition-based models that resulted from the EFA. Convergent validity is inferred if a factor’s AVE is greater than 0.5. Discriminant validity is inferred if: (1) MSV < AVE, or (2) the Square root of AVE is greater than the inter-construct correlations of latent factors on the scale (Hair et al., 2010). AVE values (see Table 2) associated with each achievement goal construct on the two models were greater than 0.5, which suggests the goal constructs exhibited convergent validity. We found no discriminant validity issues with the goal factors specified on the trichotomous model based on definition dimensions of the 3 × 2 model. However, we observed issues with discriminant validity among the separated approach and avoidance valences of each of the three goal types on the 3 × 2 model—for all but one factor, the 3 × 2 model violated both criteria for discriminant validity.

### Measurement Invariance Analysis of the Definition-Based Trichotomous Model

Finally, we examined the measurement invariance of the AGQ scale. We used the definition-based model to test for measurement invariance because we found acceptable model fit and better discriminant validity support than for the 3 × 2 model among our study sample. Researchers implicitly assume measurement invariance when they compare latent factor scores for two or more groups on a scale. However, such comparisons are meaningless if different respondent groups interpret the scale in conceptually different ways (Putnick and Bornstein, 2016). Multi-group CFA is conducted to confirm whether scale items mean the same thing to respondents across groups (Bialosiewicz et al., 2013).

We conducted measurement invariance analysis on four student groups: gender (Male or Female), college-level (Junior or Senior), race (White-American or Others), and intention to pursue graduate study (Yes or No). Four levels of invariance analysis—configural invariance, metric invariance, scalar invariance, and residual invariance—were examined for each group. Equivalency between models is typically based on chi-square difference test (Δχ^2^). However, because Δχ^2^ is rather sensitive to sample size, ΔCFI, ΔTLI, and ΔRSMEA are recommended as better alternatives (Schermelleh-Engel et al., 2003; Barrett, 2007; Meade et al., 2008; Senkbeil, 2018). Models are deemed equivalent if their ΔCFI/ΔTLI is less than | 0.01|, or ΔRSMEA is less than | 0.015| (Cheung and Rensvold, 2002; Chen, 2007).

Results of the invariance analysis showed that model fit statistics for all groups compared were better than the recommended thresholds (CFI > 0.9, TLI > 0.9, RMSEA < 0.7). We conducted invariance analyses in sequence – beginning with configural, metric, scalar, residual invariance analysis – for each group. Configural invariance, metric invariance, and scalar invariance were observed across all the groups examined, ΔCFI/ΔTLI/ΔRSMEA < | 0.01|, as reported in Table 5. Residual invariance was observed across all the groups except gender, (ΔCFI = –0.023, ΔTLI = –0.015).

**TABLE 5:** Multi-group comparison fit/measurement invariance indices for the definition-based AGQ model.

Groups	Model	χ^2^	df	CFI	TLI	RMSEA	Model Comparison			Decision
							Δ CFI	Δ TLI	Δ RMSEA	
Gender (Female, *n* = 101; Male, *n* = 249)	Configural	599.863	260	0.920	0.905	0.061	–	–	–	–
	Metric	621.516	275	0.918	0.909	0.060	–0.002	0.004	–0.001	Equivalent
	Scalar	664.074	293	0.912	0.908	0.060	–0.006	–0.001	0	Equivalent
	Residual	789.436	319	0.889	0.893	0.065	–0.023	–0.015	0.005	Not equivalent
College Level (Junior, *n* = 179; Senior, *n* = 106)	Configural	502.375	260	0.929	0.916	0.057	–	–	–	–
	Metric	521.277	275	0.928	0.920	0.056	–0.001	0.004	–0.001	Equivalent
	Scalar	545.183	293	0.926	0.923	0.055	–0.002	–0.003	–0.001	Equivalent
	Residual	589.713	319	0.921	0.924	0.055	–0.005	–0.001	0	Equivalent
Expectation of graduate school (Yes, *n* = 177; No, *n* = 159)	Configural	561.963	260	0.925	0.911	0.059	–	–	–	–
	Metric	589.752	275	0.921	0.913	0.059	–0.004	0.002	0	Equivalent
	Scalar	625.773	293	0.917	0.913	0.058	–0.004	0	–0.001	Equivalent
	Residual	684.016	319	0.909	0.913	0.059	–0.008	0.08	0.001	Equivalent
Race (White, *n* = 270; Other; *n* = 101)	Configural	599.221	260	0.933	0.921	0.056	–	–	–	–
	Metric	579.079	275	0.932	0.925	0.055	–0.001	0.004	–0.001	Equivalent
	Scalar	609.267	293	0.929	0.926	0.054	–0.003	0.001	–0.001	Equivalent
	Residual	682.620	319	0.919	0.922	0.056	–0.01	–0.004	0.002	Equivalent

*RMSEA, root mean square of approximation; TLI, Tucker Lewis Index; CFI, Comparative Fit Index; ΔCFI, change in CFI compared with the less restrictive model; ΔTLI, change in TLI compared with the less restrictive model; ΔRMSEA, change in RMSEA compared with the less restrictive model.*

### Ancillary Analysis: Group Comparisons of Achievement Goals

Following the invariance analyses, we conducted independent sample *t*-tests, and computed Cohen’s *d* of effect sizes to compare the groups on each definition-based achievement goal construct. Group comparisons may be meaningful if, at least, scalar invariance is demonstrated (Gregorich, 2006; Putnick and Bornstein, 2016). Full measurement invariance was observed across all the groups examined except gender. However, scalar invariance was established for genders.

Table 6 shows comparisons of achievement goal scores across the four groups. Female participants had significantly higher task-focused goals than male students (*p* < 0.01), and trended higher than male students on self-focused achievement goals (*p* = 0.067). Participants who reported having an intention to attend graduate school also tended to have higher task-focused goals than those with no similar intentions (*p* < 0.01). Other-based goals were significantly more prevalent among students at lower educational levels (*p* < 0.05), and those that identified themselves as White (*p* < 0.01).

**TABLE 6:** Group comparisons of achievement goal-types based on the definition-based trichotomous AGQ model.

Groups	Goal Type	Mean		*SE*	*t*	*p*-value	Cohen’s *d*
		Male *(N* = *249)*	Female *(N* = *101)*				
Gender	Task-based	25.114	26.197	0.451	2.399	0.017^**^	0.267
	Self-based	23.568	24.436	0.473	1.837	0.067^*^	0.207
	Other-based	21.824	20.889	0.626	–1.493	0.136	0.164
College level		Junior *(N* = *179)*	Senior *(N* = *106)*				
	Task-based	25.268	25.500	0.476	–0.487	0.627	0.056
	Self-based	23.537	24.016	0.493	–0.973	0.331	0.110
	Other-based	22.098	20.818	0.646	1.981	0.048^**^	0.220
Expectation of graduate school		*Yes (N* = *177)*	*No (N* = *159)*				
	Task-based	25.989	24.656	0.429	–3.112	0.002^**^	0.319
	Self-based	23.848	23.539	0.449	–0.687	0.492	0.071
	Other-based	21.868	21.156	0.595	–1.198	0.232	0.123
Race		*White (N* = *270)*	*Other (N* = *101)*				
	Task-based	25.614	24.928	0.461	1.489	0.137	0.163
	Self-based	23.719	24.081	0.484	–0.752	0.453	0.084
	Other-based	22.037	20.270	0.632	2.797	0.005^**^	0.310

^*^p < 0.010, **p < 0.05.

## Discussion and Conclusion

### Discussion of Findings

In the present study, we contrasted the 3 × 2 model of the AGQ against alternative AGO frameworks on a number of validity indicators. Consistent with previous AGQ validation studies, we observed that the 3 × 2 AGQ had a significantly better model fit than the dichotomous, trichotomous, and 2 × 2 alternative models of the AGO which were proposed and tested in earlier studies (Elliot et al., 2011; Diseth, 2015; Lower and Turner, 2016). We found that the 3 × 2 model of the AGQ had a better factor structure than the 2 × 2 and the trichotomous model variants of the scale in which task and self-referenced goals were specified together as a single factor. This observation is consistent with those reported in earlier studies and highlights that the original 3 × 2 AGO model, were task- and self-referenced goals are maintained as separate goal types, is a better conceptual representation of the achievement goal orientation framework based on the 3 × 2 AGQ (Elliot et al., 2011; Diseth, 2015). By implication, the finding also supports theoretical arguments for conceptualizing task-referent and self-referent goal types as separate components of mastery achievement goals (Elliot et al., 2011).

On the one hand, the 3 × 2 model of the scale was slightly structurally better than the definition-based model we specified based on our EFA results. On the other hand, we observed very high correlations between the approach and avoidance valences of the three goal types (0.99 ≤ *r* ≤ 0.86). Several other prior studies have reported finding similar high correlations between approach and avoidance goals of the AGQ (Elliot et al., 2011; Murayama et al., 2011; Johnson and Kestler, 2013; Diseth, 2015). However, we envisage that observation of rather high inter-factor correlations between approach and avoidance goal types of the 3 × 2 AGQ across multiple studies warrants the need to further explore the constancy of the scale to discriminate between approach and avoidance goal across different learners and learning contexts. Some have argued that very high inter-factor correlation could indicate that such highly correlated factors may be measuring the same construct (Brown, 2006; Kline, 2010; Cohen et al., 2013). In addition to high inter-factor correlations, AVE and MSV values for the goal factors of the 3 × 2 model strongly indicated the AGQ scale may have lacked the sensitivity needed to differentiate between approach and avoidance valences of each goal type for the participants in our study sample.

Most prior factor-analytic studies of the AGQ have been based on CFA (Murayama et al., 2011) – which means that a 3 × 2 AGO framework was imposed on the AGQ scale items, *a priori*. However, the CFA technique is less sensitive to detecting cross-loadings between factor indicators because models are fixed based on theory (Brown, 2006). On the contrary, EFA technique is data-driven, and reveals underlying factor structure between scale items without any constraint of a potential theoretical bias. Murayama et al. (2011) compared an EFA outcome in which performance achievement goal indicators (both approach and avoidance valenced) were allowed to freely load against another EFA in which performance achievement goal indicators were all forced to load unto one factor. They found that performance-avoidance and performance-approach goal indicators loaded on two separate factors when items were allowed to freely load. They also found that the two-factor model had a better structural fit than when all the performance goal indicators where constrained to load together.

Contrary to their observation, we found no similar support for separate approach and avoidance valenced task-, self-, and other-referenced achievement goals in our study. Rather, an unconstrained EFA of the AGQ items yielded three factors in which the approach and avoidance indicators of each goal type loaded together. Even after specifying a six-factor model in a subsequent EFA, the AGQ items did not load a 3 × 2 framework that was consistent with theory. Furthermore, we found better indications of convergent and discriminant validity, and internal reliability for a three-factor model of the 3 × 2 AGQ scale based only on a definition dimension compared to that of the actual 3 × 2 model of the scale.

A potential explanation for the lack of distinctions between approach and avoidance goals for the three goal types is that items on the 3 × 2 AGQ are not sufficiently sensitive to capture the uniqueness of approach and avoidance goals in all learners or learning contexts. The correlations observed between these factors in our sample lie on the upper end of what has been reported in past studies, similar to Johnson and Kestler (2013) and Lower and Turner (2016); whereas Elliot et al. (2011) and Diseth (2015) reported more distinctive goal valences in their studies. The approach taken by León-del-Barco et al. (2019) to use second-order factors for achievement goal orientations, may be useful to explain variation in responses on valenced questions in other work.

Invariably, it is possible that factors unique to our study context may have accounted for the lack of indistinct goal valences observed among our participants. Some have suggested that achievement goal valences may be less distinct in certain student demography at different developmental stages. For example, Bong (2009) suggested that younger students may be less cognitively aware, and may not differentiate between approach and avoidance goal types. However, how consistently older students exhibit and distinguish approach and avoidance goal types across different learning contexts is less understood. In the same vein, approach and avoidance goal types are less visible among students of certain ethnic backgrounds (Murayama et al., 2009).

Whether scale factors are distinctive is often an important methodological consideration when evaluating the validity of multi-dimensional latent factors (Hilkenmeier et al., 2020). A recurring observance of high correlations between approach and avoidance valences across multiple studies could imply that the 3 × 2 AGQ items does not always capture distinct approach and avoidance achievement goal valences across mutiple samples and contexts. Contrarywise, it could also be that students goal behaviors are less valenced in certain learning contexts (Diseth, 2015). In essence, the result of this study either highlights that achievement goal valences are more or less salient in different sample demography or learning contexts, or that the 3 × 2 AGQ scale does not always capture the approach-avoidance dichotomy in some learners. Hence, more studies are needed to further investigate how salient achievement goal valences are across different contexts.

Lastly, our findings validated a prior report on the measurement invariance of the AGQ questionnaire. Elliot et al. (2011) conducted multi-group analysis and established that the 3 × 2 AGQ had measurement invariance across samples of German and American undergraduate students. We examined whether the definition-based model was invariant across gender, race, college-level, and intention of our study participants to attend graduate school. We found strict invariance in each of these group comparisons except gender – which assures that there are no differential item functioning across the different comparison groups (Putnick and Bornstein, 2016). It also indicates that items on the scales have similar conceptual meaning across diverse groups. The observed measurement invariance result supports the use of the scale for exploring achievement goal orientation differences across groups. More also, the pattern of achievement goal differences we observed among participant groups in our study may be explored further in future studies.

### Implication of Findings for Future Research

The foregoing observations highlight some important considerations for researchers who might intend to use the AGQ scale in achievement goal-related research. The 3 × 2 model of the AGQ scale had a better structural validity than alternative models. However, AVE and MSV indicators suggested that the 3 × 2 framework might lack strong discriminant validity, as our data suggested. This might explain why high approach-avoidance inter-factor correlations has been observed across multiple studies (Johnson and Kestler, 2013; Lower and Turner, 2016). Notably, high inter-factor correlations between approach and avoidance goal valences, as observed in this and other studies, could cause multicollinearity problems and undermine inferences drawn when the 3 × 2 achievement goal model is used in regression or structural model-based studies. Whether researchers combine approach and avoidance valences of the different achievement goal types of the 3 × 2 AGQ or use them as separate valenced goals ultimately depends on their research objectives. Based on our observations however, we would rather suggest that they carefully consider how choosing to separate or combine approach and avoidance goals could affect the result of their analysis, and the inferences that can be made from such analysis. When the AGQ is used, it may be worthwhile to first examine the structure fit of participants’ responses before making decisions to separate or combine approach and avoidance goal factors.

Second, our major observation in this study (i.e., to combine the approach and avoidance valences of the 3 × 2 AGQ scale) is at variance with the theoretical proposition of the 3 × 2 AGO framework. However, it draws attention to the need to further explore when and how goal valences manifest across different learners and learning contexts. Future studies may further explore how often the 3 × 2 AGQ scale captures approach and avoidance goal valences across learners and learning contexts, or whether learners even recognize different goal valences in different learning contexts.

Third, we observed strong or strict measurement invariance across different groups of participants in our study. This result suggests that the 3 × 2 AGQ scale items are understood to mean the same thing to the different participant groups in our study, which supports earlier findings about the invariance of the scale (Elliot et al., 2011). This observation also means that groups can be meaningfully compared using achievement goal scores based on the 3 × 2 AGQ scale. Group differences that were observed to be significant in this study (Table 6) could be further explored in future studies to better understand achievement goal behaviors across different learner categories. For example, future research should examine the achievement goal behaviors of students who nurture graduate school ambitions. Perhaps such an investigation could provide insights in mentoring undergraduate students for graduate school. Future studies could also explore how achievement goal behaviors evolve over students’ academic careers.

### Limitations and Conclusion

A limitation of our study is that our findings may not generalize across a broader population due to the limited, and less diverse students sample that the study was based on. The limited sample scope was due to our intention to investigate the AGO in a discipline (i.e., engineering) that is notably different from those sampled in prior validations of the 3 × 2 AGQ. Studies that draw on more diverse student demograph could further explore the robustness or conditionality of the 3 × 2 AGQ across different learners and learning contexts. Similarly, studies that draw on a larger and more diverse student demography will be necessary to strengthen existing measurement invariance evidence of the AGQ scale.

In the present study, we explored and evaluated different validity and reliability artifacts for the 3 × 2 framework and a definition-based alternative model of the AGQ scale. We found a better statistical fit for the 3 × 2 model relative to other competing achievement goal models. Despite the structure fit of the 3 × 2 model however, we observed multiple artifacts in support of a definition-based model of the scale that combines the approach and avoidance valences of the three achievement goal types. First, there were very high correlations between avoidance and approach valences of task, self, and other goal types of the 3 × 2 AGQ model. Second, an EFA of the AGQ showed that the 3 × 2 goal factors converged as three definition-based goal types. Third, evaluation of AVE-based validity showed that the three goal factors on a definition-based model of the scale had better validity and reliability properties than the six factors of a 3 × 2 model of the scale.

In summary, while we found good structural support for a 3 × 2 configuration of the AGQ, a definition-based model of the scale – where the approach and avoidance goal indicators were combined – had better validity and reliability properties in this study. On the one hand, it could be that the 3 × 2 AGQ scale does not always capture distinct goal valences across different learners or learning context. One the other hand, it is also possible that students do not always exhibit or report valenced achievement goal types in all learning contexts. In conclusion, as researchers evaluate the tradeoffs of their methodological and theoretical positions, we recommend they first evaluate the validity and reliability implications of separating or combining goal valences when the 3 × 2 AGQ scale is used in predictive models that integrate achievement goal orientation theory in their research studies.

## Data Availability Statement

The raw data supporting the conclusions of this article will be made available by the authors, without undue reservation.

## Ethics Statement

Ethical review and approval was not required for the study on human participants in accordance with the local legislation and institutional requirements. The patients/participants provided their written informed consent to participate in this study.

## Author Contributions

AO was involved in data collection, analysis, and manuscript writing. AJ and OO was involved in the manuscript writing. All the authors contributed to the article and approved the submitted version.

## Conflict of Interest

The authors declare that the research was conducted in the absence of any commercial or financial relationships that could be construed as a potential conflict of interest.

## Publisher’s Note

All claims expressed in this article are solely those of the authors and do not necessarily represent those of their affiliated organizations, or those of the publisher, the editors and the reviewers. Any product that may be evaluated in this article, or claim that may be made by its manufacturer, is not guaranteed or endorsed by the publisher.
